# Radial head prosthesis: results overview

**DOI:** 10.1007/s12306-017-0492-x

**Published:** 2017-08-14

**Authors:** E. Carità, A. Donadelli, L. Cugola, P. Perazzini

**Affiliations:** Clinica San Francesco, Via Monte Ortigara 20, 37120 Verona, Italy

**Keywords:** Radial head prosthesis, Radial head fractures, Humeroradial post-traumatic arthritis, Radial head replacement, Elbow stiffness

## Abstract

**Background/purpose:**

Radial head replacement is frequently used in treatment of radial head fractures or sequela. Impossibility to restore a correct anatomy, acute elbow traumatic instability and failure of osteosynthesis hardware are the most common indications. The authors describe their case studies and results on the implantation of various radial head prostheses.

**Materials:**

Between June 2005 and June 2016, 28 radial head prostheses were implanted in the same number of patients with an average follow-up of 49 months (6–104). Indications for implantation were: Mason type III and IV radial head fractures and post-traumatic arthritis due to failure of previous treatments. Monopolar prostheses were used and were press-fit implanted via Kaplan’s lateral access and Kocher’s anconeus approach to the humeroradial joint. At the follow-up, assessments were made of the pain, according to the visual analogic scale, range of motion (ROM), stability and functionality according to the Mayo Elbow Performance Score, presence of osteolysis and mobilization during radiography tests, personal satisfaction of the patients, Disabilities of the Arm, Shoulder and Hand and Patient-Rated Wrist Evaluation outcomes measurements.

**Results:**

At the follow-up, we recorded an average level of pain of 1.8 in patients under acute treatments for radial head fractures and a marked reduction in the remaining cases from 6.7 to 2.1. ROM was found on average to be 107° of flexion–extension and 159° of pronosupination. Personal satisfaction was good–excellent in 23 cases. There was no case of infection; removal of the implant was necessary in three cases due to mobilization of the stem and oversized implants. In six cases, bone resorption was seen at the level of the prosthetic collar and it was in all cases asymptomatic.

**Conclusions:**

The results of this study suggest that the use of prostheses, if well positioned, is a valid solution in the treatment of secondary arthritis and fractures of the radial head with poor prognosis, with good results in the reduction of pain, recovery of movement and improved quality of life.

## Introduction

The humeroradial joint is the lateral column of the elbow and is an important stabilizer for axial and valgus loading [1].

Its integrity ensures good stability of the elbow even in the presence of other lesions, such as lesion of the medial collateral ligament (MCL) or minor coronoid fractures.

Fractures of the radial head constitute 1.7–5.4% of upper limb fractures and 33–75% of all elbow fractures [2] and are often associated with complex lesions that also affect the medial compartment or coronoid.

According to studies by the Mayo Foundation, the MCL is the primary stabilizer of the elbow joint and the radial head is second in importance in the stabilization during loading and valgus stress [3].

Therefore, from the biomechanical point of view, in the presence of injury of the MCL, coronoid fracture or lesions of the lateral collateral ulnar ligament (LUCL), the radial head is considered a structure of fundamental importance [4].

In many works, the results and limits of radial head excision are described in the treatment of complex fractures that lead, in the long term, to valgus instability, longitudinal instability with positive ulnar variance and pain in the wrist, lack of strength and the appearance of ulnohumeral degenerative changes [5, 6].

It is by now agreed that the indications for radial head excision are isolated comminuted fractures of the radial head without signs of longitudinal or medial instability in elderly or low demanding patients [7–11].

In the presence of complex fractures of the radial head, even a well-performed osteosynthesis can result in a malunion or a painful or stiff elbow due to bone resorption, loosening, mobilization of the hardware.

Two recent prospective randomized trials have demonstrated improved outcomes in radial head prosthesis compared to osteosynthesis (ORIF) for complex unstable fractures, with a greater frequency of complications in ORIF, such as premature failure of the synthesis and non-union [12, 13], and one study determines three fragments to be the cut-off number in order to proceed with prosthesis implant as the preferred treatment [14].

The use of the radial head prosthesis was a much-debated subject in the past due to numerous reports of a high percentage of complications and repeat interventions to review or remove the implant [15, 16].

Recent anatomical and biomechanical studies have enabled the reasons for the previous failures to be understood and the critical points of the surgical technique and the anatomical references for positioning a good implant to be identified, thereby reducing complications to a minimum.

Moreover, the creation of osteointegrative and biocompatible materials and increasingly anatomical prosthetic profiles has enabled the adaptability and tolerance of the implants to be improved, reducing the conflict with the humerus and increasing its potential duration.

In this work, the authors describe their experience in the implant of the radial head prosthesis in Mason type III and IV fractures and in the outcomes of radial fractures treated conservatively or by ORIF that resulted in malunion or were associated with joint pain and stiffness.

## Materials and methods

Between June 2005 and June 2016, 31 radial head prostheses were implanted in as many patients. In the follow-up, 28 patients were assessed. The average age of the patients was 49 years old (18–71) and the male/female ratio was 12/16.

Fractures were classified using the Mason classification [17].

Indications for the implant were Mason type III and IV radial fractures and post-traumatic arthritis in outcomes of radial head fracture with stiffness and pain.

Patients who simultaneously presented other fractures of the elbow joint requiring osteosynthesis and patients treated surgically for associated medial instability were excluded from the review.

In all, 19 patients suffering from fractures and nine patients suffering from post-traumatic arthritis were treated.

The prostheses were press-fit implanted by lateral access to the humeroradial joint in accordance with Kaplan or using Kocher’s anconeus approach.

During surgery, the integrity of the annular ligament and the radial collateral ligaments were assessed. The annular ligament was always repaired, when it could be reconstructed, and the radial collateral ligaments were repaired or reinserted with sutures again if avulsed or detached for implantation of the prosthesis or surgical approach. Any fractures of the tip of coronoid were treated by excision, cerclage of the tip or stabilization with Kirschner wires. Patients with other kind of coronoid fracture were excluded.

In all cases, cementless and monopolar prostheses manufactured by Acumed and Tornier (Mophic) were used with anatomical (Acumed) or pyrocarbon (Mophic) head.

Post-operatively, the elbow was immobilized in an articulated elbow guard for 3–5 days at 90° leaving the pronosupination free, and subsequently mobilized between 30° and 120° up to the 20th day, later freeing the flexion and extension up to 0° and retaining the elbow guard for the first 30 days.

During the first 5 days, patients underwent physical therapy to reduce edema of the limb and pericicatricial tissue; in the following days, physical therapy was helpful for passive and active articular recovery and the reinforcement of the extensor muscles; the recovery and muscular reinforcement of biceps and triceps in flexion–extension of the elbow was begun only after 45 days.

At the follow-up, assessments were made of the pain, according to the visual analogic scale (VAS), the range of motion (ROM), the stability and functionality according to the Mayo Elbow Performance Score (MEPS), the presence of osteolysis and mobilization of the implant during radiographic tests, the personal satisfaction of the patients according to four grades (excellent, good, sufficient, poor), the Disability of Arm Shoulder and Hand (DASH) score and the Patient-Rated Wrist Evaluation (PRWE) score.

## Results

The results were gathered on 28 patients with an average follow-up of 49 months (6–118).

**Fig. 1:**
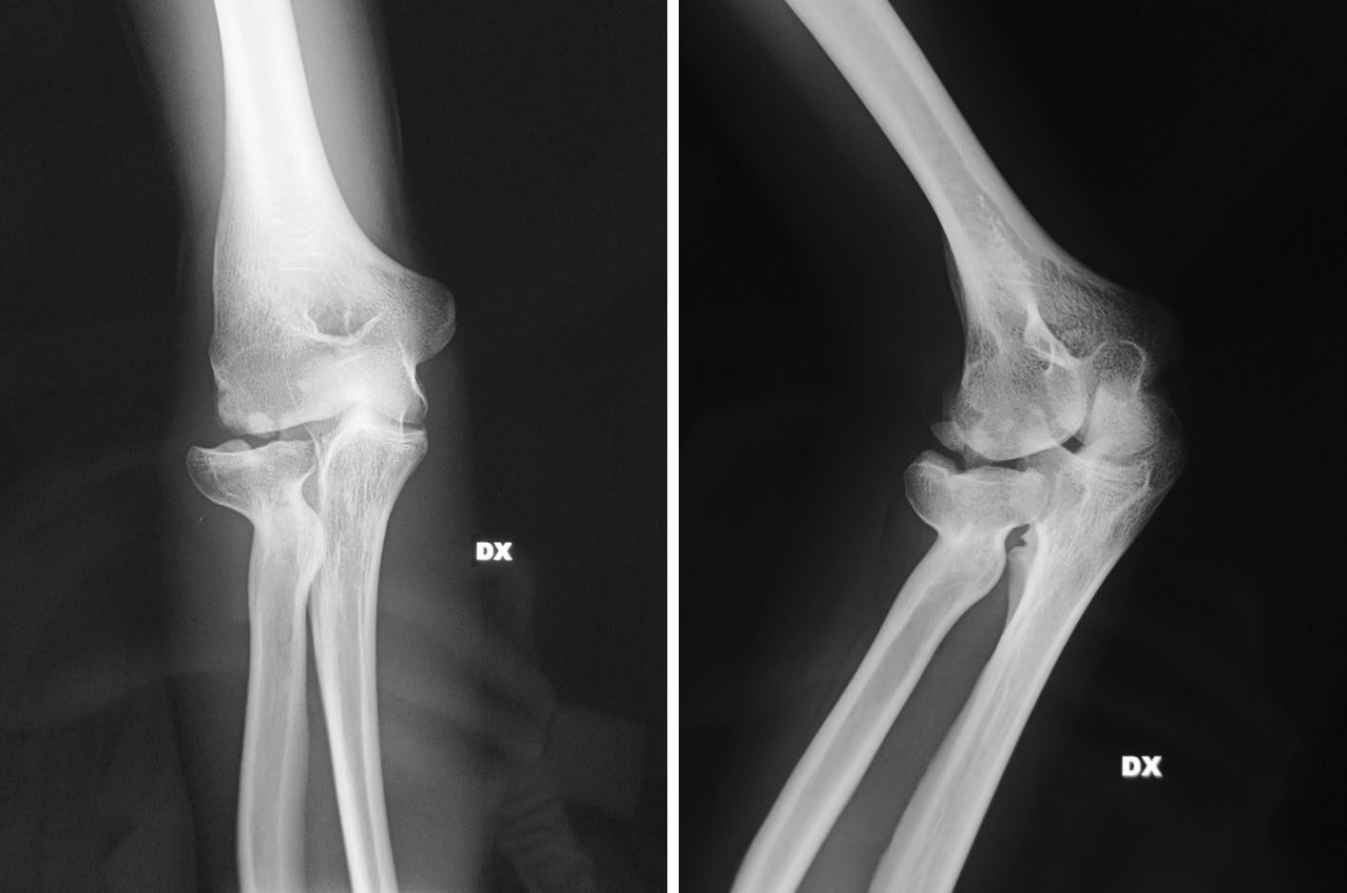
​Pre-op X-rays of 18-year-old boy with radial head malunion, calcifications and secondary humeroradial arthritis

**Fig. 2:**
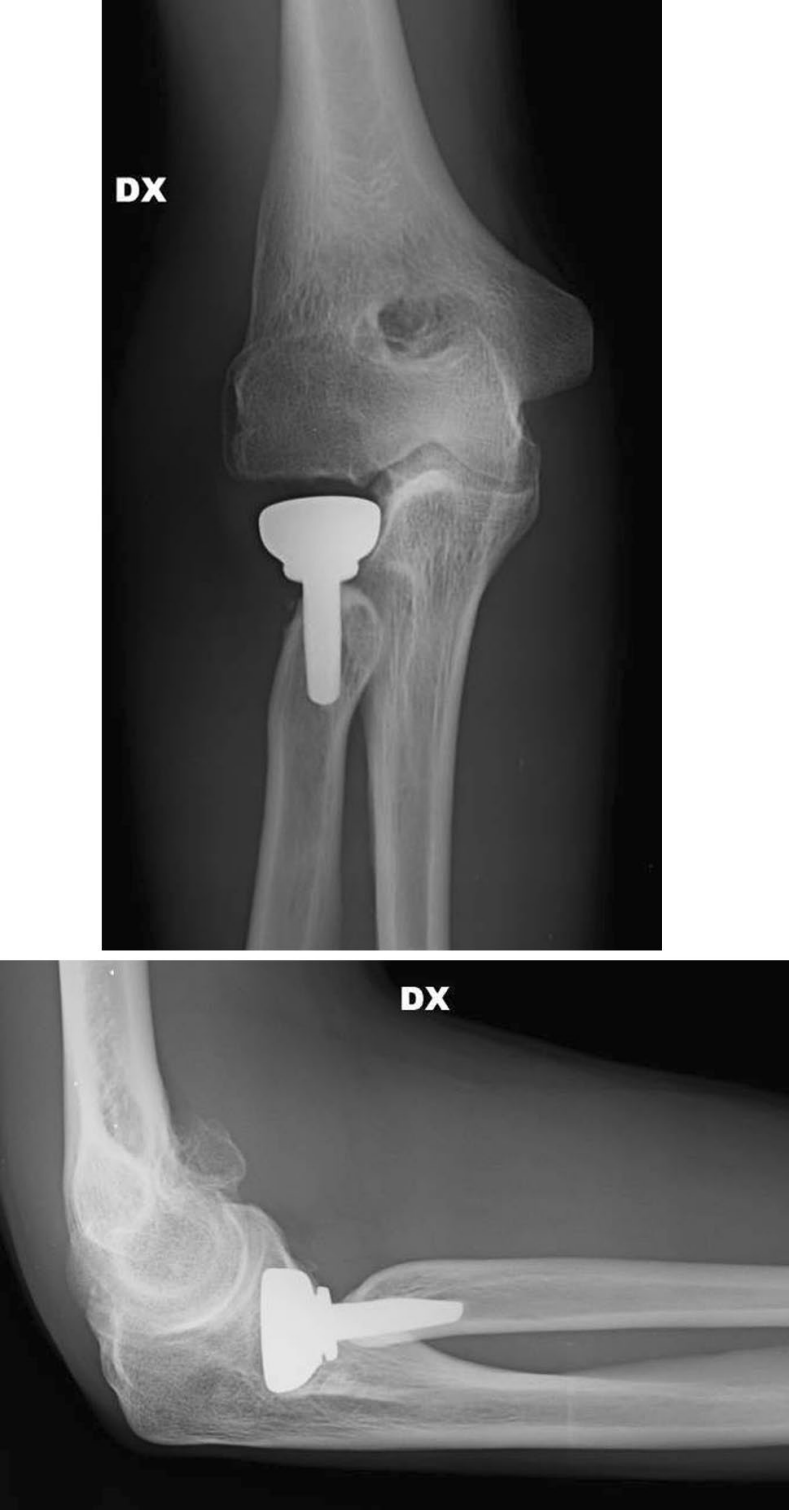
Five-year X-ray follow-up with good position of implant and stress shielding radial neck resorption

**Fig. 3:**
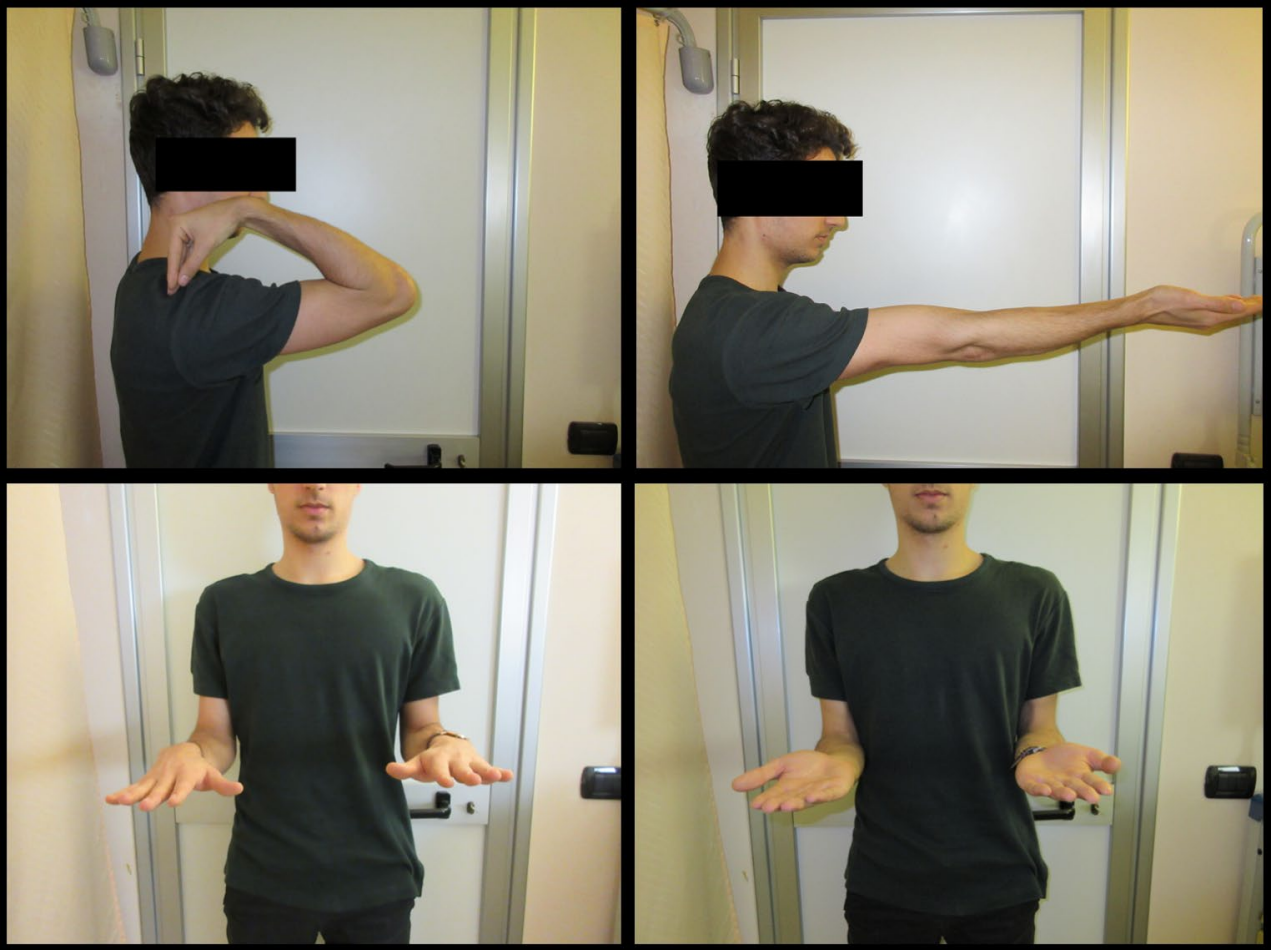
Five-year clinical follow-up showing maintenance of total ROM recovery

At the follow-up, we recorded an average level of pain of 1.8 in patients under acute treatments for fractures of the radial head and a reduction in the remaining cases from 6.7 to 2.1. The ROM was found on average to be 107° of flexion–extension and 159° of pronosupination. Stability was good in 25 cases, and the MEPS was 89 (Figs. 1, 2, 3).

**Fig. 4:**
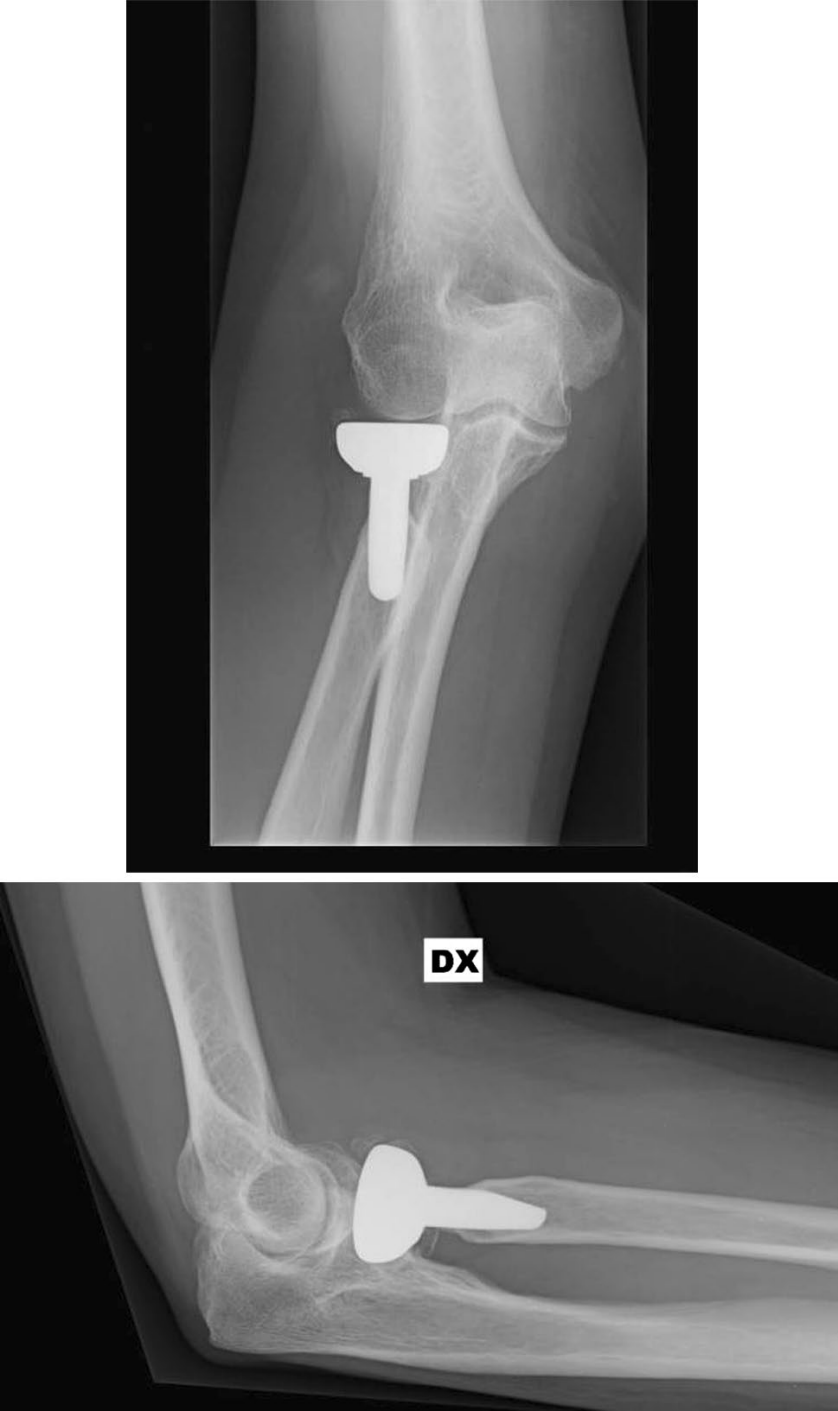
48 months X-ray follow-up of the same patient showing stress shielding radial neck resorption

**Fig. 5:**
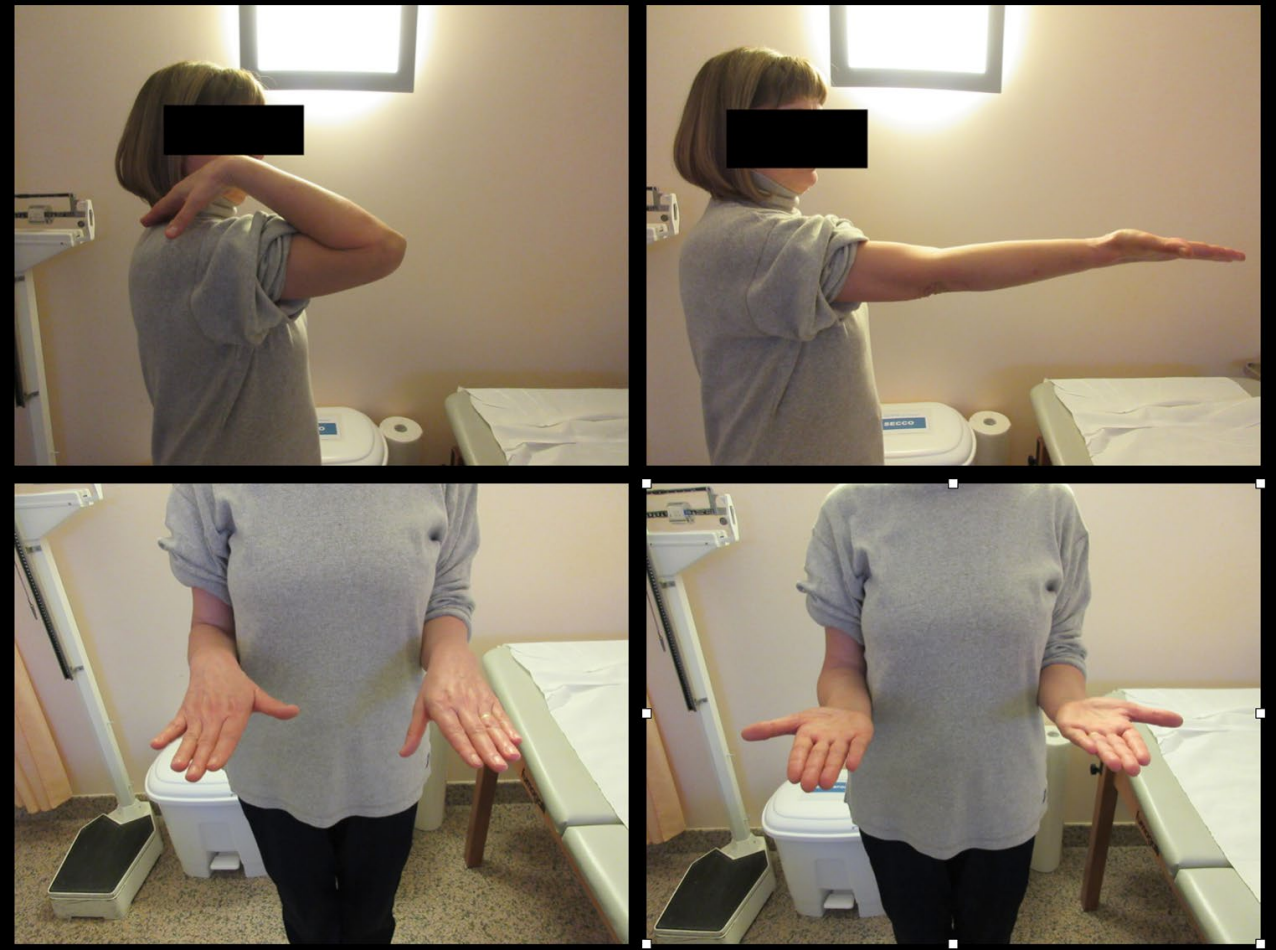
48 months clinical follow-up showing good ROM recovery

Radiological examination revealed one case of osteolysis and mobilization of the stem, one case of erosion of the capitellum surface due to overstuffing, six asymptomatic cases of resorption of the neck of the radius at the level of the prosthetic collar from stress shielding (Figs. 4, 5) and two cases of heterotopic periprosthetic ossification. In three cases, the removal of an implant was necessary, in one case due to mobilization of the stem and in two because of pain caused by an oversized implant.

Personal satisfaction was good–excellent in 14 cÐases, good in nine cases, sufficient in two cases and poor in three cases.

The DASH and PRWE scores at the follow-up stood at 14, 2 and 29.

## Discussion

The literature describes various options for the treatment of fractures of the radial head, including ORIF, radial head resection or prosthesis [18] but, in most traumas and fractures, where it is important to reconstruct the radial head and the radio-humeral contact, the choice is between osteosynthesis and replacement.

From the anatomical-vascular point of view, radial epiphysis is entirely contained inside the articular capsule and the vascularization of a radial head in an adult is delegated to a series of intraosseous vessels that run vertically from the neck of the radius up to the radial head [19]. Yamaguchi [20] showed how a vessel directly vascularizes the radial head by entering from the non-articular anterolateral part of the neck. Consequently, a fracture of the neck can devascularize the epiphysis.

For this reason, in the presence of comminution or severe dislocation of the fracture fragments, as in Mason type III and IV fractures, even a successful osteosynthesis can often result in osteonecrosis of the fragments, pseudoarthrosis, mobilization or failure of the hardware generating a stiff, unstable or painful elbow [21, 22].

In these cases, the surgical solution involves radial head excision or prosthetization.

There are three critical points during implant of radial head prosthesis: the stability of the stem, the size of the head, the height of the head and the stability of the humeroradial joint.

**Fig. 6:**
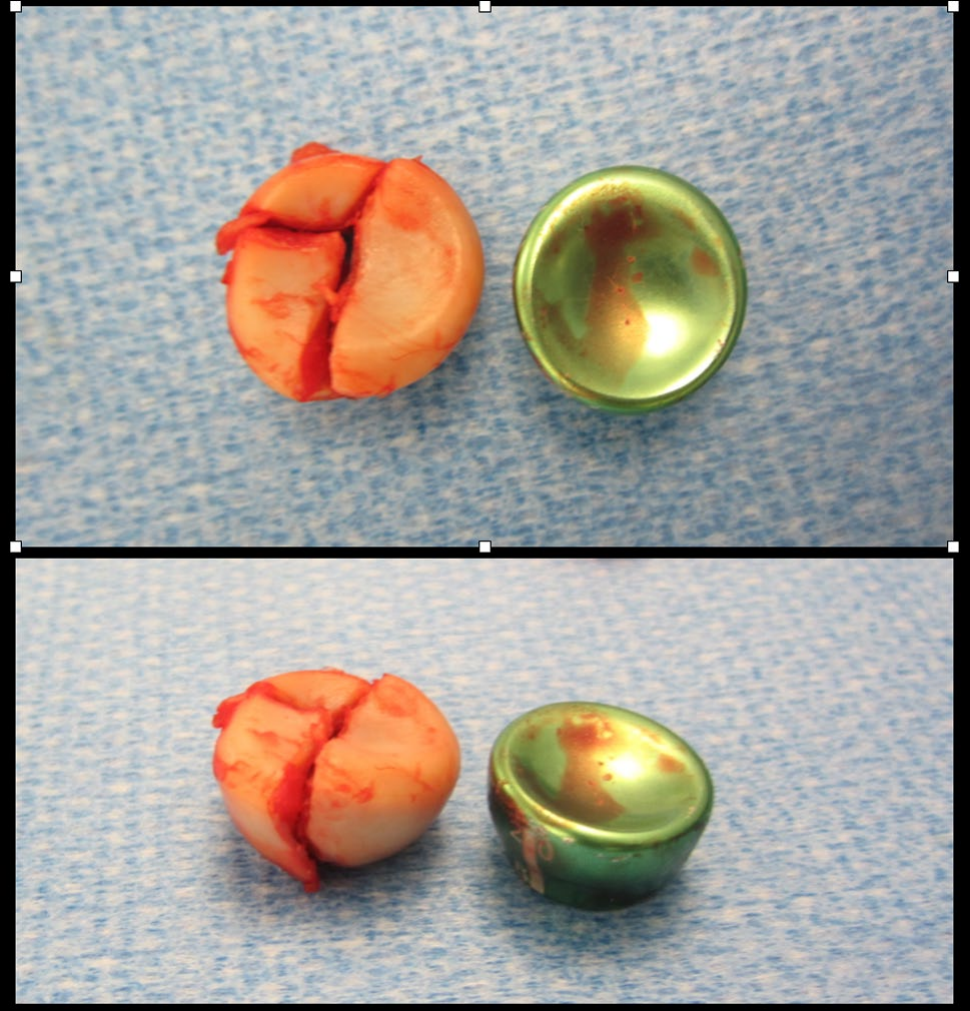
Intraoperative statement of prosthesis dimensions after radial head removal for comminuted fracture

**Fig. 7:**
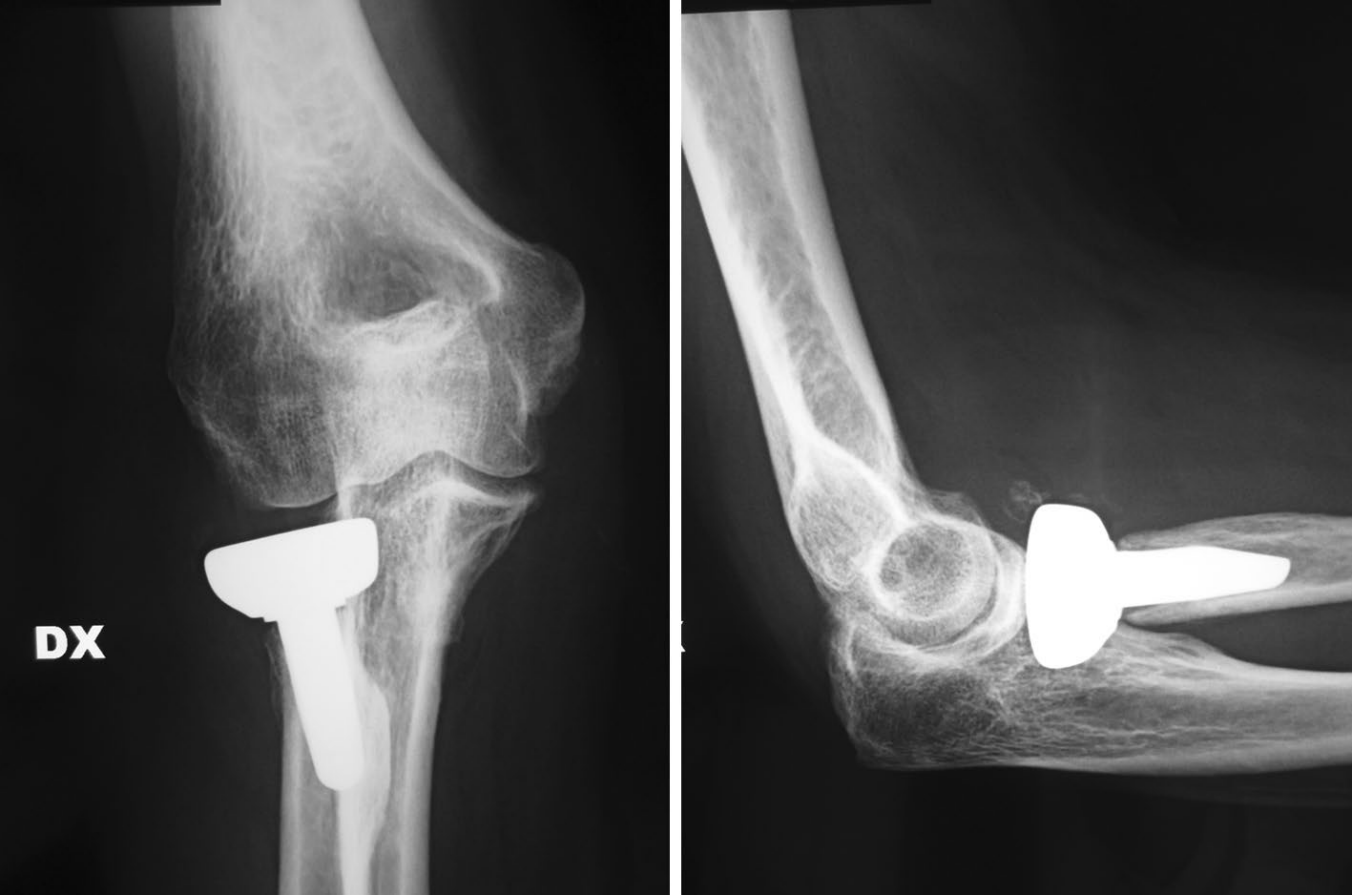
Two months X-ray follow-up of radial head prosthesis implanted for radial head malunion

Taking care over these steps determines the outcome of the intervention. During implantation, it is therefore important to obtain a good fit of the stem, which must be the bigger and as stable as possible to reduce the risk of mobilization and cut out [23] measure the diameter of the removed radial head if possible and position a smaller prostheses equal to the existing capitulum [24] (Fig. 6) obtain a good match between the height of the head and the sigmoid notch of the ulna [25, 26] respect the landmark given by the coronoid in the lateral and anteroposterior projections and do not exceed its height proximally as regards the height of the prosthesis [27] (Fig. 7), check the integrity of the radial ligamentous complex and where necessary restore or reconstruct it to ensure good stability of the implant and the joint.

The results obtained as regards articulation, pain and the MEPS can be superimposed on those in the literature [28, 29], while we reported a very low revision rate of around 10% (3 cases out of 29). In one case, the revision was due to the stem being too small, which caused its mobilization; in two other cases, it was due to excessive length of the implant, with clinical and radiological signs of overstuffing.

Overstuffing and pain from oversized prostheses are the most frequent causes of failure and review of the implant, registering 10–15% [16–30].

In our case study, the percentage stands at around 6% and regards the most dated cases, positioned at the start of the authors’ learning curve.

A systematic review reports revision rates of between 0 and 29% statistically irrespective of the type of prostheses (monopolar or bipolar), and the material it is made of and the type of positioning (press-fit or cement) [31].

Duckworth et al. describe 29 out of 105 cases of revision due to: stiffness 12, painful loosening 5, isolated pain 4, subluxation 3, synovitis 2, ulnar nephritis 2, infection 1 [16].

Ha et al. [32] describe radiographic causes of failures in 62 out of 258 implants: heterotopic ossification 53.2%, stiffness or pain due to tension and thickening of the synovial or capsular tissue 43.5% and infection 3.2%.

As regards the resorption of the collar from stress shielding (Figs. 4, 5), the six cases reported come within the average given in the literature, where up to 63% of implants with such radiographic evidence are described. This phenomenon presents with periosteal bone resorption starting from the 7th month post-op, which usually stabilizes after the 15th month. The resorption is not destabilizing and does not lead either to failure or the revision of the implant [33].

One limit of the study is the retrospective analysis, another is the number of cases that can be increased and another is the use of two different types of implants with different anatomical features of the radial head and of the stems.

Another limit is the simultaneous assessment of fractures under acute treatment and traumatic outcomes that assume the presence of already traumatized or scarred tissue with reduced elasticity and recovery capacity compared to tissues under acute treatments.

## Conclusions

The medium-long term results of the prostheses implanted on fractures or painful outcomes of fractures of the radial head are comparable with those present in the literature and show that the result can be maintained over time by respecting the implant positioning criteria.

Radial head prosthesis is therefore a suitable option for fractures of the radial head that cannot be reduced or synthetized or in cases of malunion with secondary arthritis in the outcomes of conservative or surgical treatment of fractures.
